# Recombinant Hepatitis E Viruses Harboring Tags in the ORF1 Protein

**DOI:** 10.1128/JVI.00459-19

**Published:** 2019-09-12

**Authors:** Dagmara Szkolnicka, Angela Pollán, Nathalie Da Silva, Noémie Oechslin, Jérôme Gouttenoire, Darius Moradpour

**Affiliations:** a Division of Gastroenterology and Hepatology, Centre Hospitalier Universitaire Vaudois, University of Lausanne, Lausanne, Switzerland; University of Southern California

**Keywords:** HEV, open reading frame 1 protein, positive-strand RNA virus, replicase, replication complex, replicon, transposon, viral life cycle

## Abstract

Hepatitis E virus (HEV) is one of the most common causes of acute hepatitis and jaundice in the world. Current understanding of the molecular virology and pathogenesis of hepatitis E is incomplete, due particularly to the limited availability of functional tools. Here, we report the development of tagged HEV genomes as a novel tool to investigate the viral life cycle. A selectable subgenomic HEV replicon was subjected to random 15-nucleotide sequence insertion using transposon-based technology. Viable insertions in the open reading frame 1 (ORF1) protein were selected in a hepatoblastoma cell line. Functional insertion sites were identified downstream of the methyltransferase domain, in the hypervariable region (HVR), and between the helicase and RNA-dependent RNA polymerase domains. HEV genomes harboring a hemagglutinin (HA) epitope tag or a small luciferase (NanoLuc) in the HVR were found to be fully functional and to allow the production of infectious virus. NanoLuc allowed quantitative monitoring of HEV infection and replication by luciferase assay. The use of HA-tagged replicons and full-length genomes allowed localization of putative sites of HEV RNA replication by the simultaneous detection of viral RNA by fluorescence *in situ* hybridization and of ORF1 protein by immunofluorescence. Candidate HEV replication complexes were found in cytoplasmic dot-like structures which partially overlapped ORF2 and ORF3 proteins as well as exosomal markers. Hence, tagged HEV genomes yield new insights into the viral life cycle and should allow further investigation of the structure and composition of the viral replication complex.

**IMPORTANCE** Hepatitis E virus (HEV) infection is an important cause of acute hepatitis and may lead to chronic infection in immunocompromised patients. Knowledge of the viral life cycle is incomplete due to the limited availability of functional tools. In particular, low levels of expression of the ORF1 protein or limited sensitivity of currently available antibodies or both limit our understanding of the viral replicase. Here, we report the successful establishment of subgenomic HEV replicons and full-length genomes harboring an epitope tag or a functional reporter in the ORF1 protein. These novel tools should allow further characterization of the HEV replication complex and to improve our understanding of the viral life cycle.

## Abstract (precis)

Hepatitis E virus (HEV) infection is an important cause of acute hepatitis and may lead to chronic infection in immunocompromised patients. Knowledge of the viral life cycle is incomplete due to the limited availability of functional tools. In particular, low levels of expression of the ORF1 protein or limited sensitivity of currently available antibodies or both limit our understanding of the viral replicase. Here, we report the successful establishment of subgenomic HEV replicons and full-length genomes harboring an epitope tag or a functional reporter in the ORF1 protein. These novel tools should allow further characterization of the HEV replication complex and to improve our understanding of the viral life cycle.

## INTRODUCTION

Hepatitis E virus (HEV) infection is one of the most common causes of acute hepatitis and jaundice in the world (1, 2). Human-pathogenic HEV strains are classified in the *Orthohepevirus* genus within the *Hepeviridae* family. HEV genotypes 1 and 2 are transmitted from human to human and can cause large, primarily waterborne outbreaks in resource-limited settings (3). HEV genotypes 3 and 4 represent a primarily porcine zoonosis in middle- and high-income areas (4). HEV genotype 3 (and, to a lesser extent, genotypes 4 and 7) can persist in immunocompromised patients, mainly organ transplant recipients, and can cause chronic hepatitis and cirrhosis. In addition, HEV genotype 3 is an important cause of neurological and other extrahepatic manifestations (5, 6). A vaccine is currently available only in China, and antiviral therapy is limited to the use of ribavirin or pegylated alpha interferon. Hence, HEV represents a global health challenge where better understanding of viral life cycle may translate into improved management in the future.

HEV is a positive-strand RNA virus that circulates in the blood in a quasi-enveloped form, i.e., wrapped in cellular membranes, and that is excreted as a nonenveloped particle in stool (7–9). Its 7.2-kb RNA genome comprises 3 open reading frames (ORF) that are translated into (i) the ORF1 protein, representing the replicase and harboring methyltransferase, helicase, and RNA-dependent RNA polymerase (RdRp) activities; (ii) the ORF2 protein, corresponding to the viral capsid; and (iii) the ORF3 protein, a small, palmitoylated membrane protein which plays an important role in virion secretion (10, 11).

Although certain functions of the ORF1 protein such as those involving methyltransferase, helicase, and RdRp have been well studied, there is controversy as to whether and by what mechanism the ORF1 polyprotein may be processed during the HEV life cycle (7–9). What is more, the detection of ORF1 protein in cells replicating subgenomic replicons or infected with HEV has been notoriously difficult due to the low levels of expression and/or limited sensitivity of currently available antibodies (Abs) (12, 13), hindering characterization of the subcellular localization of HEV RNA replication sites and of the composition of viral replication complexes.

Tagging with different epitopes, fluorescent proteins, or reporter enzymes has been widely used to visualize and characterize viral proteins (14). Although these tools are powerful, the highly compact genomes of positive-strand RNA viruses do not generally tolerate random insertions. Therefore, we combined transposon-mediated random insertion with selection in a subgenomic replicon system to identify sites in the HEV ORF1 protein that would be amenable to tagging in a functional context and allow visualization of the viral replicase. This approach identified viable insertion sites downstream of the methyltransferase domain, in the hypervariable region (HVR), and between the helicase and RdRp domains. HEV genomes harboring a hemagglutinin (HA) epitope tag or a small luciferase (nanoluciferase [NanoLuc]) in the HVR were found to be fully functional and to allow the production of infectious virus. HA-tagged replicons and full-length genomes allowed localization of putative sites of HEV RNA replication by the simultaneous detection of RNA by fluorescence *in situ* hybridization (FISH) and of ORF1 protein by immunofluorescence. Candidate HEV replication complexes were found in cytoplasmic dot-like structures which partially overlap ORF2 and ORF3 proteins as well as exosomal markers. Hence, use of these tagged genomes should allow further investigation of the structure and composition of the HEV replication complex.

## RESULTS

### Identification of functional insertion sites in the HEV ORF1 protein.

Transposon-mediated random insertion was combined with selection in a subgenomic replicon system to identify sites in the HEV ORF1 protein that would allow tagging in a functional context and visualization of the viral replicase. HEV genotype 3 strain 83-2-27 (15) was used for this purpose, as, in contrast to the commonly used Kernow-C1/p6 strain (16), it does not harbor any naturally acquired insertion in the HVR.

**FIG 1:**
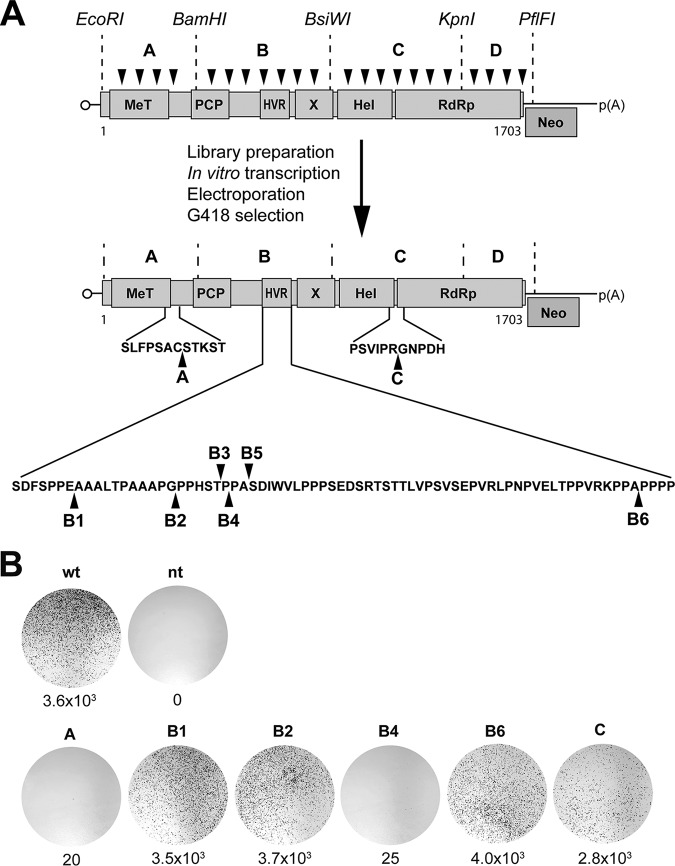
Identification of functional insertion sites in HEV ORF1 protein. (A) Schematic outline of the transposon-based random insertion screen. The selectable subgenomic HEV replicon is represented with ORF1 domains designated methyltransferase (MeT), papain-like cysteine protease (PCP), hypervariable region (HVR), macrodomain (X), helicase (Hel), and RNA-dependent RNA polymerase (RdRp). Neo, neomycin phosphotransferase. MuA transposase-mediated random insertion was carried out on pUC-HEV83-2_Neo, followed by *in vitro* transcription of sublibraries A to D, electroporation into Hep293TT cells, and selection with G418. Sequencing identified transposon insertions at sites A, B1 to B6, and C, as denoted by the black arrowheads. Sequences are detailed in Table 1. (B) Replication efficiency of subgenomic HEV replicons harboring transposon insertions. Hep293TT cells were electroporated with *in vitro*-transcribed RNA from the parental pUC-HEV83-2_Neo replicon construct (wild type [wt]) or subgenomic replicons harboring transposon insertions. Nontransfected cells (nt) served as a control. After G418 selection, colonies were fixed and stained with crystal violet. Results from a representative experiment are shown, with the number of colonies per microgram of transfected RNA indicated below each plate.

A neomycin-selectable subgenomic HEV 83-2-27 replicon (HEV83-2-27_Neo) was subjected to random insertion of transposons bearing a kanamycin resistance gene (Fig. 1A). Four HEV ORF1 fragments defined by unique restriction sites and designated fragments A to D were cloned from the library into the parental replicon, followed by excision of the kanamycin resistance cassette. The four replicon sublibraries harboring 15-nucleotide random insertions in different regions of ORF1 were transcribed *in vitro*, followed by electroporation of replicon RNA into Hep293TT human hepatoblastoma cells and selection with G418. Hep293TT cells were used for this purpose given their good tolerance to electroporation and capacity to produce HEV, as described previously (11). RNA from surviving cell populations was reverse transcribed, cDNAs encompassing the ORF1 fragments were cloned into the pCR-BluntII-TOPO vector, and the resulting plasmids were sequenced.

Sequencing revealed 8 viable insertion sites in the ORF1 protein: 1 located in fragment A, downstream of the methyltransferase domain (identified in 8 clones); 6 located in fragment B, within the HVR (identified in 1 to 3 clones each); and 1 located in fragment C, between the helicase and RdRp domains (identified in 4 clones) (Table 1; see also Fig. 1A). No viable insertion was identified in fragment D. Hence, all sequenced insertions mapped to the same sites downstream of the methyltransferase or between the helicase and RdRp domains whereas several viable sites were identified in the HVR and none in the RdRp domain.

**TABLE 1:** Positions of the 15-nucleotide transposon insertions identified in ORF1 of the HEV-83-2-27 clone^*a*^

Insertion	Location (nt)	Nucleotide sequence	Amino acid sequence
A	1361–1362	GCCTGC**TGCGGCCGCACCTGC**TCTACA	AC**CGRTC**ST
B1	2597–2598	CCTGAG**TGCGCGCGCACTGAG**GCAGCT	PE**CGRTE**AA
B2	2630–2631	CCAGGT**GCGGCCGCACCAGGA**CCACCT	PG**AAAPG**PP
B3	2645–2646	TCTACT**GCGGCCGCATCTACT**CCACCC	ST**AAAST**PP
B4	2648–2649	ACTCCA**TGCGGCCGCACTCCA**CCCGCT	TP**CGRTP**PA
B5	2654–2655	CCCGCT**ATGCGGCCGCACGCT**AGTGAT	PA**MRPHA**SD
B6	2786–2787	CCAGCT**GCGGCCGCACCAGCA**CCACCT	PA**AAAPA**PP
C	4133–4134	CCTCGT**GTGCGGCCGCATCGT**GGTAAC	PR**VRPHR**GN

a Transposon sequences are bold and underlined. See Fig. 1A for a graphic representation of insertion sites. nt, nucleotide.

As insertion sites B3, B4, and B5 were very closely spaced, we chose to further examine only the B4 site. To confirm the functionality of the identified insertion sites, the fragments harboring the insertions were cloned into the parental HEV83-2-27_Neo replicon, followed by *in vitro* transcription, electroporation of replicon RNA into Hep293TT cells, and G418 selection. As shown in Fig. 1B, all identified insertion sites were found to be viable. However, the levels of replication efficiency differed significantly, being comparable to the parental replicon for sites B1, B2, and B6 (3.5 to 4.0 × 10^3^ colonies per μg RNA), slightly lower for site C (2.8 × 10^3^ colonies per μg RNA), and lower by 2 orders of magnitude for sites A and B4 (20 to 25 colonies per μg RNA).

### HEV replicons harboring an HA tag in the HVR allow visualization of the ORF1 protein.

**FIG 2:**
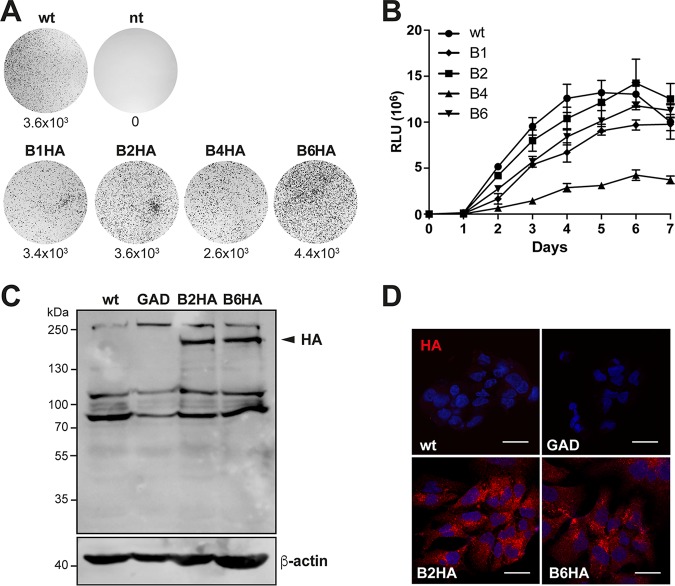
Replication of HA-tagged subgenomic HEV replicons. (A) Replication efficiency of HA-tagged subgenomic HEV replicons. Hep293TT cells were fixed 12 days postelectroporation with *in vitro*-transcribed RNA from a subgenomic replicon construct (pUC-HEV83-2_Neo_B1-HA, pUC-HEV83-2_Neo_B2-HA, pUC-HEV83-2_Neo_B4-HA, or pUC-HEV83-2_Neo_B6-HA) and stained with crystal violet. The parental HEV83-2-27_Neo replicon (wild type [wt]) and nontransfected cells (nt) served as positive and negative controls, respectively. Results from a representative experiment are shown, with the number of colonies per microgram of transfected RNA indicated below each plate. (B) Culture supernatants from Hep293TT cells electroporated with *in vitro*-transcribed RNA from the parental pUC-HEV83-2_Gluc (wild type [wt]) or the pUC-HEV83-2_Gluc-derived replicon construct harboring 15-nucleotide transposon insertions in site B1, B2, B4, or B6 were analyzed for luciferase activity over 7 days. Gaussia luciferase activity was measured by adding 10 μl of supernatant from Hep293TT cells electroporated with luciferase HEV replicon constructs to 60 μl of substrate buffer (0.8 μM coelenterazine–PBS). Relative light units (RLU) were determined four times for each time point. (C and D) Immunoblotting (C) and immunofluorescence detection (D) of HA-tagged ORF1 protein in Hep293TT cells. Cells were electroporated with wild-type (wt), polymerase-defective (GAD), or HA-tagged (B2HA and B6HA) selectable subgenomic replicon constructs. (C) Protein lysates were prepared 5 days postelectroporation and separated by 8% SDS-PAGE, followed by sequential immunoblotting using rabbit MAb C29F4 against HA and mouse MAb AC-15 against β-actin. The arrowhead denotes HA-tagged ORF1 protein. Molecular weight markers are indicated on the left. (D) Cells were fixed 5 days postelectroporation and subjected to immunofluorescence with rabbit MAb C29F4 against HA as primary antibody and Alexa Fluor 594 anti-rabbit IgG as secondary antibody. Cell nuclei were counterstained with DAPI. The scale bar represents 20 μm.

In order to visualize the ORF1 protein and, thereby, candidate replication complexes, we introduced an HA epitope tag into the identified functional insertion sites within the HVR of subgenomic replicon HEV83-2-27_Neo. To increase accessibility of the epitope tag, the HA sequence was flanked on both sides by 5-amino-acid (aa) sequences derived from the original transposon (see Materials and Methods for details). As shown in Fig. 2A, subgenomic replicons harboring an HA tag in the B1, B2, and B6 sites replicated as efficiently as the parental replicon (3.4 to 4.4 × 10^3^ colonies per μg RNA) whereas the insertion at the B4 site was slightly less well tolerated (2.6 × 10^3^ colonies per μg RNA). Of note, replicons harboring an HA tag in the A and C sites did not replicate or replicated at low levels only (0 and 5.3 × 10^2^ colonies per μg RNA, respectively; data not illustrated), suggesting that these sites were unable to tolerate or tolerated insertions of greater than 15 nucleotides or 5 amino acids only poorly.

To confirm and extend the findings described above, we cloned the insertions within the HVR into a subgenomic replicon allowing monitoring of Gaussia luciferase as a reporter. As shown in Fig. 2B, clones B2 and B6 showed replication dynamics similar to those seen with the parental replicon (B2, 1.4 × 10^7^; B6, 1.2 × 10^7^; wild type [wt], 1.3 × 10^7^ relative light units [RLU] at day 6 posttransfection) whereas clones B1 and B4 showed lower replication efficiency. Hence, the scope of our further studies was narrowed to insertion sites B2 and B6.

Electroporation of Hep293TT cells with B2HA and B6HA subgenomic replicon RNAs allowed detection of the HA-tagged ORF1 protein by immunoblotting (Fig. 2C) and immunofluorescence (Fig. 2D). As shown in Fig. 2C, only unprocessed ORF1 protein of approximately 190 kDa was observed by immunoblotting, suggesting the lack of polyprotein processing in this genuine replication system. Immunofluorescence revealed a cytoplasmic dot-like staining pattern for the HA-tagged ORF1 protein expressed by B2HA and B6HA replicons, while, as expected, no signal was detected in cells electroporated with the parental replicon or a replication-defective control. Importantly, immunoblotting and immunofluorescence analyses using polyclonal antibodies against the methyltransferase and helicase domains did not allow conclusive visualization of ORF1 protein under the same conditions (data not illustrated).

### HA-tagged full-length HEV genomes are infectious.

**FIG 3:**
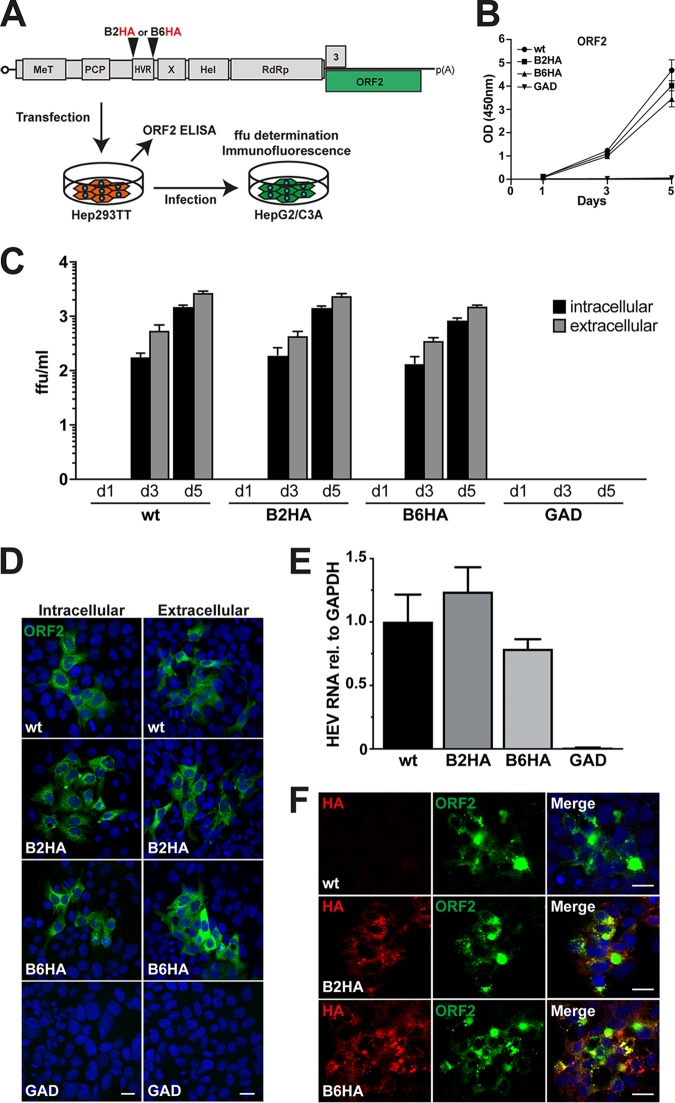
Infectivity of HA-tagged full-length HEV genomes. (A) Schematic representation of the HEV genome (see Fig. 1 legend for abbreviations) and of the experimental workflow. Hep293TT cells were transfected with *in vitro*-transcribed RNA from full-length HEV 83-2-27 constructs. Levels of ORF2 protein in culture supernatants were measured by ELISA. Culture supernatants as well as cell lysates were used to infect naive HepG2/C3A cells, followed by determination of focus-forming units (ffu), quantitative RT-PCR, and immunofluorescence analyses. (B) HEV ORF2 release from electroporated Hep293TT cells was measured by ELISA. The *y* axis represents optical density (OD) values at 450 nm after blank subtraction. Error bars represent means ± standard deviations (SD) of triplicate measurements for each condition. (C) Culture supernatants and cell lysates were collected at day 1, 3, and 5 postelectroporation and used to infect naive HepG2/C3A cells, followed by determination of focus-forming units (ffu) as described in Materials and Methods. Infectivity was determined for parental (wild type [wt]), HA-tagged (B2HA or B6HA), and polymerase-defective (GAD) full-length genomes using MAb 1E6 against ORF2 protein. Means ± SD of results from 6 replicates for each condition are shown. (D) HepG2/C3A cells infected with either intracellular or extracellular virus samples were fixed at day 5 and subjected to immunofluorescence using MAb 1E6 against ORF2 protein followed by confocal analysis. Nuclei were counterstained with DAPI. The scale bars represent 20 μm. (E) HepG2/C3A cells inoculated with cell lysates obtained from Hep293TT cells transfected with parental (wild-type [wt]), B2HA, B6HA, or polymerase-defective (GAD) full-length genomes were analyzed 5 days postinfection by quantitative RT-PCR. Samples were normalized to GAPDH RNA, and results are expressed as relative expression levels compared to the parental genome. Error bars represent means ± SD of results from six replicates for each condition. (F) Immunofluorescence detection of HA epitope tag and ORF2 protein after infection. HepG2/C3A cells infected with wild-type (wt) or HA-tagged HEV (B2HA or B6HA) from Hep293TT cell lysates were subjected to immunofluorescence using rabbit MAb C29F4 against HA and mouse MAb 1E6 against ORF2 protein, followed by appropriate secondary antibodies (red and green, respectively). Cell nuclei were counterstained with DAPI (blue). The scale bar represents 20 μm.

Efficient replication of the B2HA and B6HA replicons encouraged us to examine whether HA-tagged full-length genomes are able to produce infectious viral particles. Hence, the insertions were cloned into full-length HEV 83-2-27 constructs, followed by *in vitro* transcription and electroporation of full-length viral RNA into Hep293TT cells (Fig. 3A). Levels of HEV ORF2 protein released into culture supernatants were analyzed by enzyme-linked immunosorbent assay (ELISA) at days 1, 3, and 5 posttransfection. As shown in Fig. 3B, the levels of viral capsid release were comparable between the parental and B2HA as well as B6HA genomes (*P* > 0.1 at day 5). Supernatants and cell lysates harvested at days 1, 3, and 5 postelectroporation were used to infect HepG2/C3A cells, representing a particularly permissive subclone of human hepatoblastoma cell line HepG2 (17, 18). As shown in Fig. 3C and in the representative immunofluorescence analyses shown in Fig. 3D, extracellular virus and intracellular virus collected from Hep293TT cells replicating the full-length B2HA and B6HA genomes showed infectivity similar to that seen with the parental virus (*P* > 0.1).

To further examine the infectivity of HA-tagged recombinant viruses, HepG2/C3A cells were infected with intracellular virus collected from Hep293TT cells replicating the full-length B2HA and B6HA genomes, followed by analysis of cell lysates by quantitative reverse transcription-PCR (RT-PCR) and immunofluorescence 5 days postinfection. Our results revealed similar HEV RNA levels in cells infected with parental or HA-tagged B2HA or B6HA viruses (*P* = 0.07 or 0.13, respectively) (Fig. 3E), as confirmed by ORF2 protein detection by immunofluorescence (Fig. 3F).

Hence, viruses harboring an HA tag in the ORF1 protein were found to be infectious, with infectivities and replication efficiencies comparable to those of the parental virus. Importantly, the HA tag remained detectable for at least 25 days of cell passaging and sequence analyses revealed stable maintenance of the HA tag insertion over the course of this extended observation period (data not shown; cf. Fig. 8C).

### HA-tagged full-length HEV genomes allow visualization of putative replication sites.

**FIG 4:**
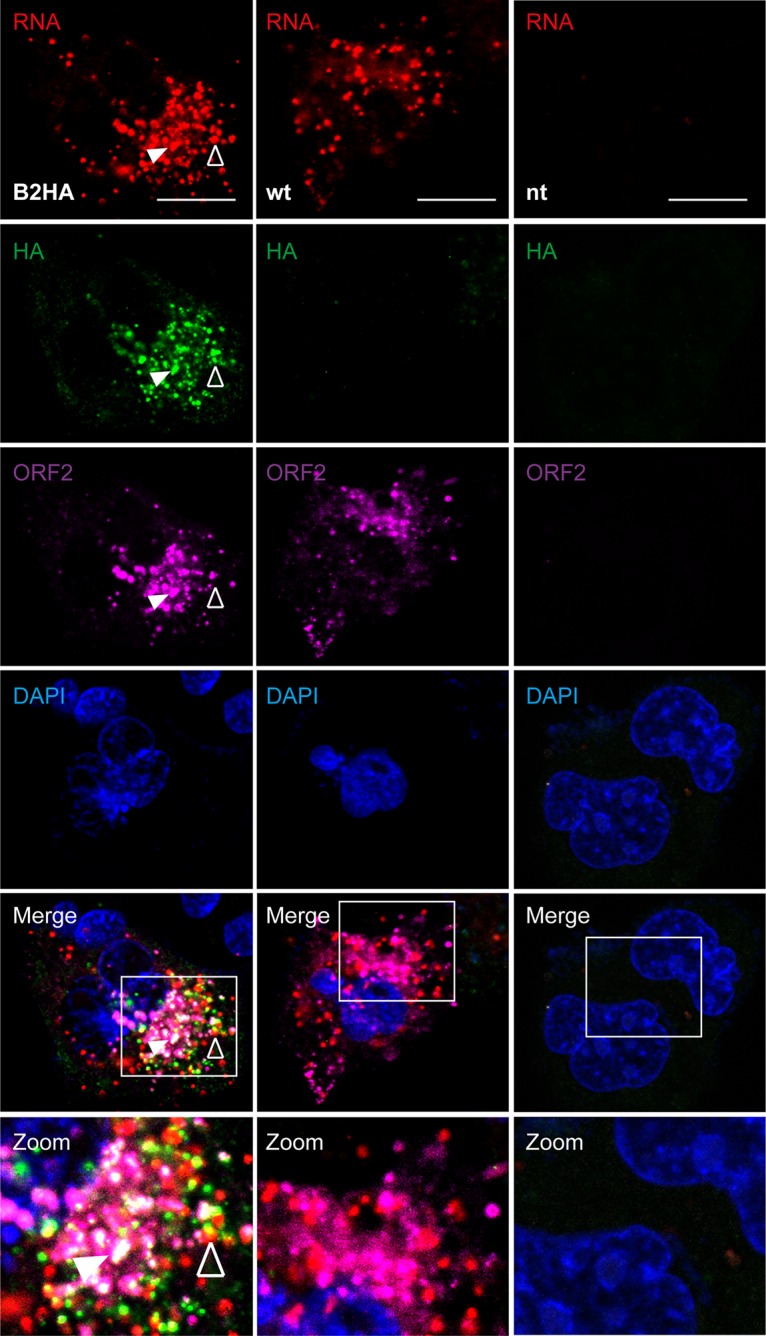
Simultaneous detection of HEV RNA and HA-tagged ORF1 protein in cells replicating full-length genomes. Hep293TT cells transfected with full-length B2HA or wild-type (wt) HEV RNA were fixed 5 days posttransfection and subjected to fluorescence *in situ* hybridization (FISH) using the HEV-specific probe V-HEV-C2 (red), followed by immunofluorescence detection of HA-tagged ORF1 protein using rabbit MAb C29F4 against HA (green) and mouse MAb 1E6 against ORF2 protein (magenta). Cell nuclei were counterstained with DAPI (blue). Nontransfected cells (nt) served as negative control. White squares indicate fields shown in higher magnification at the bottom. The empty arrowhead denotes an example of HEV RNA and ORF1 protein colocalization while the filled arrowhead denotes colocalization of HEV RNA as well as ORF1 and ORF2 proteins. The scale bar represents 20 μm.

As stated in the introduction, the difficulty of visualizing ORF1 protein in an authentic replication or infection context limited investigation of the subcellular site of HEV RNA replication. Therefore, we explored whether HA-tagged full-length HEV genomes would allow visualization of putative replication sites. To this end, we examined HEV RNA and ORF1 protein by FISH coupled with immunofluorescence in Hep293TT cells replicating full-length HEV genomes. Five days posttransfection, cells were fixed and processed for FISH using an HEV-specific probe, signal amplification, and binding of fluorescent probes, followed by immunofluorescence. As shown in Fig. 4, full-length HEV83-2_B2HA RNA appeared in cytoplasmic dot-like structures which partially colocalized with the HA-tagged ORF1 protein. The occurrence of overlapping signals for viral RNA and the replicase points to putative sites of HEV RNA replication (empty arrowhead). Moreover, ORF2 protein partially colocalized with the viral RNA and HA-tagged ORF1 (filled arrowhead). Analogous results were obtained with construct HEV83-2_B6HA (data not illustrated).

**FIG 5:**
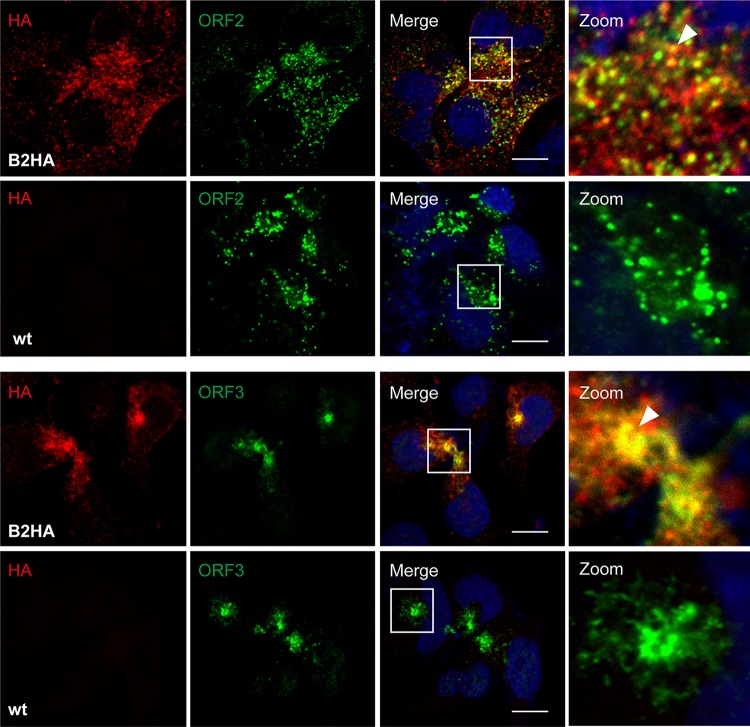
HA-tagged ORF1 protein partially colocalizes with ORF2 and ORF3 proteins. Hep293TT cells transfected with full-length B2HA or wild-type (wt) HEV RNA were fixed 5 days posttransfection and subjected to immunofluorescence using rabbit MAb C29F4 against HA (red) and either mouse MAb 1E6 against ORF2 or mouse recombinant MAb MRB198 against ORF3 protein (green). Cell nuclei were counterstained with DAPI (blue). White squares indicate fields shown in higher magnification on the right. Arrowheads denote examples of ORF1 protein colocalization with ORF2 or ORF3 protein. The scale bar represents 20 μm.

Studies involving yeast two-hybrid and coimmunoprecipitation assays in heterologous expression systems identified interactions between the macrodomain (X domain) of ORF1 protein and the ORF3 protein (19) as well as between the ORF2 and ORF3 proteins (20). To further examine potential interactions between structural and nonstructural proteins in an authentic replication system, we electroporated Hep293TT cells with *in vitro*-transcribed full-length HEV83-2_B2HA RNA and performed immunofluorescence analyses using antibodies against the HA tag and ORF2 or ORF3 proteins. As shown in Fig. 5, confocal laser scanning microscopy revealed partial colocalization of HA-tagged ORF1 protein with ORF2 and ORF3 proteins, suggesting that replication and assembly sites are closely connected.

### Subcellular localization of candidate HEV replication complexes.

Formation of a replication complex composed of viral proteins, replicating viral RNA, rearranged cellular membranes, and other host factors is a hallmark of positive-strand RNA viruses (21). The results described above localize candidate HEV replication complexes to dot-like structures in the cytoplasm. To gain further insight into their subcellular localization, we performed double-label immunofluorescence analyses of Hep293TT cells replicating full-length HEV83-2_B2HA RNA using antibodies against the HA tag and markers for different subcellular compartments, including protein disulfide isomerase (PDI) for the endoplasmic reticulum (ER), ERGIC-53 for the ER-Golgi intermediate compartment, GM130 for the Golgi apparatus, and mitochondrial antiviral signaling protein (MAVS) for mitochondria as well as the tetraspanins CD63 and CD151 for exosomes.

**FIG 6:**
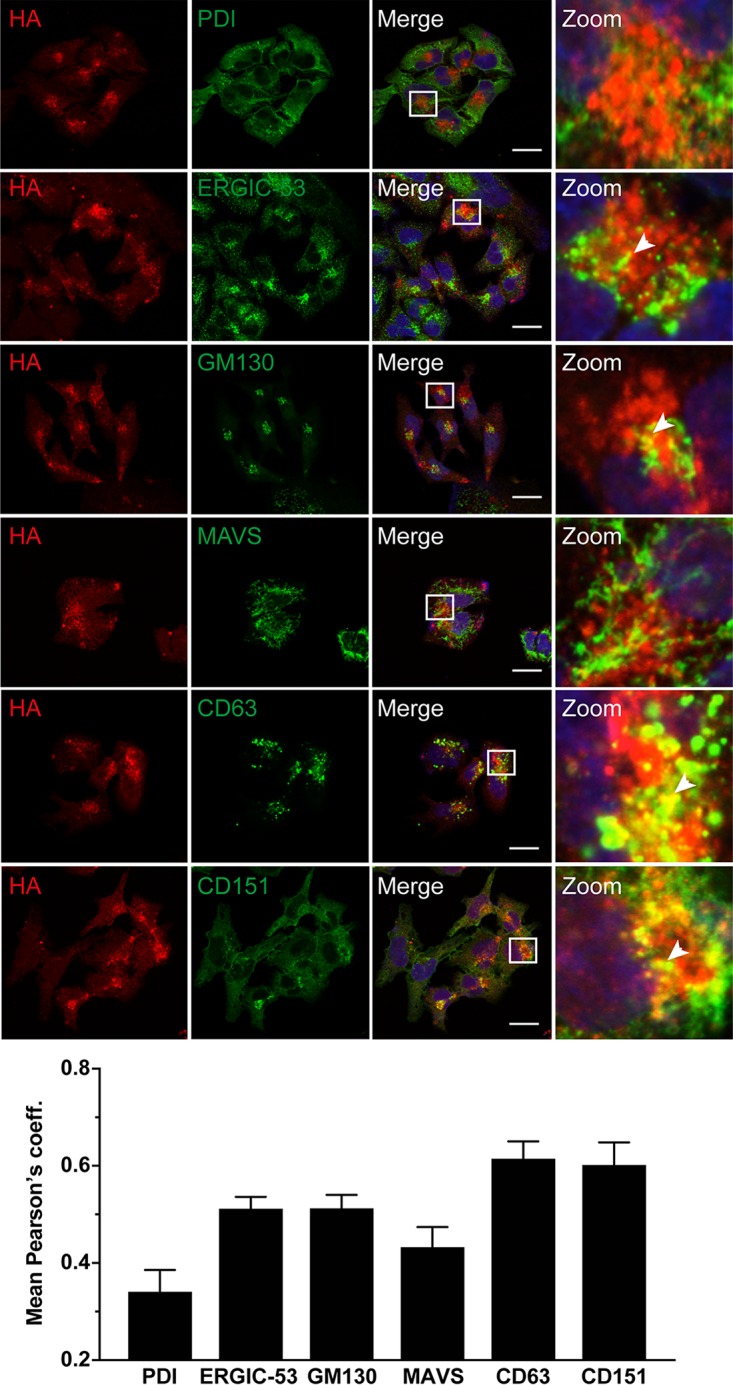
HA-tagged ORF1 protein partially colocalizes with exosomal markers. Hep293TT cells transfected with full-length B2HA or parental (wild type [wt]) HEV RNA were fixed 5 days posttransfection and subjected to immunofluorescence using rabbit MAb C29F4 against HA (red) and antibodies against markers for different cell compartments (green), including protein disulfide-isomerase (PDI) for the endoplasmic reticulum (ER), ERGIC-53 for the ER-Golgi intermediate compartment, GM130 for the Golgi apparatus, MAVS for mitochondria, and the tetraspanins CD63 and CD151 for exosomes (see Materials and Methods for abbreviations and the list of antibodies used). Cell nuclei were counterstained with DAPI (blue). White squares indicate fields shown in higher magnification on the right. Arrowheads denote examples of colocalization. The scale bar represents 20 μm. Means ± SEM of Pearson's correlation coefficients (coeff.) determined for each condition (*n* ≥ 10 cells each) are represented as histograms in the bottom panel.

As shown in Fig. 6, ORF1 protein revealed by the HA tag colocalized best with CD63 and CD151 as markers of exosomes, with mean Pearson's coefficients of 0.61 ± 0.11 and 0.60 ± 0.15, respectively. Minimal colocalization was observed with markers of the ER-Golgi intermediate compartment and the Golgi apparatus (mean Pearson's coefficients, 0.51 ± 0.08 and 0.51 ± 0.09, respectively). However, there was no colocalization with markers for the ER or mitochondria (mean Pearson's coefficients, 0.34 ± 0.13 and 0.43 ± 0.09, respectively).

### HEV ORF1 protein is membrane associated.

**FIG 7:**
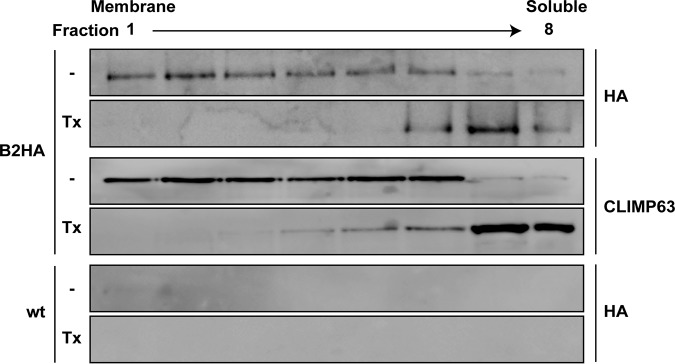
HA-tagged ORF1 protein is membrane-associated. Hep293TT cells electroporated with full-length B2HA or parental (wild type [wt]) HEV RNA were lysed in a hypotonic buffer, followed by membrane flotation as described in Materials and Methods. Lysates were not treated (−) or treated with 1% Triton X-100 (Tx) prior to gradient centrifugation. Eight fractions were collected from the top and analyzed by immunoblotting using rabbit MAb C29F4 against the HA tag. Detection of CLIMP63 served as a control for an integral membrane protein.

Given the partial colocalization of HA-tagged ORF1 protein with exosomal membrane proteins CD63 and CD151, we investigated a potential membrane association of ORF1 protein by membrane flotation analyses. Hep293TT cells replicating full-length HEV83-2_B2HA were lysed in a hypotonic buffer, followed by equilibrium centrifugation in Nycodenz gradients. Under these conditions, membrane proteins float to the upper, low-density fractions, as exemplified by CLIMP63, an integral ER membrane protein (22) (Fig. 7). As shown in Fig. 7, HA-tagged ORF1 protein was found mainly in the membrane fractions and disruption of membranes by the use of 1% Triton X-100 resulted in a shift to the lower, high-density fractions. These results indicate that the HEV replicase is associated with cellular membranes.

### NanoLuc insertion allows quantitative monitoring of HEV replication.

**FIG 8:**
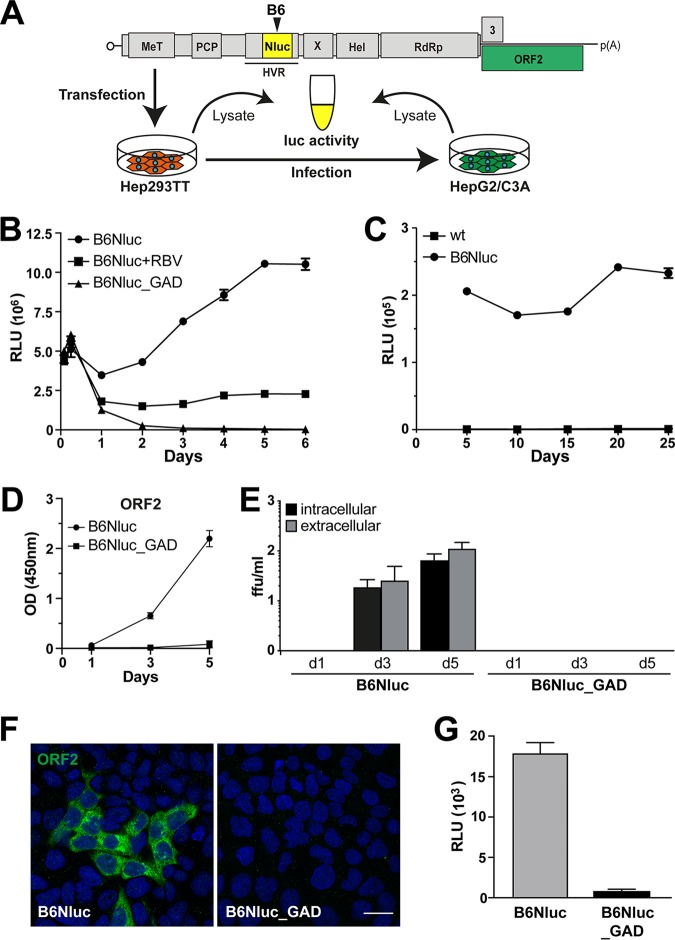
HEV harboring a luciferase gene within ORF1 allows quantitative monitoring of viral replication. (A) Schematic representation of the HEV genome (see Fig. 1 legend for abbreviations) and of the experimental workflow. (B) Hep293TT cells were transfected with *in vitro*-transcribed RNA from full-length HEV 83-2-27 constructs with a NanoLuc insertion in ORF1 (B6Nluc). The antiviral activity of ribavirin (RBV) was evaluated at a concentration of 50 μM. A polymerase-defective mutant (B6Nluc_GAD) served as negative control. NanoLuc activity was measured at 2 and 6 h as well as at 1 to 6 days posttransfection. Mean relative light units (RLU) ± SD from triplicate experiments measured twice each are shown. (C) NanoLuc activity was measured in Hep293TT cells electroporated with parental (wild type [wt]) or B6Nluc full-length RNA over 25 days (5 passages). Each data point was obtained from 3 independent samples normalized to one million cells each. (D) HEV ORF2 protein release from Hep293TT cells electroporated with B6Nluc or B6Nluc_GAD full length RNA was measured by ELISA. The *y* axis represents the OD values at 450 nm after blank subtraction. Error bars represent means ± SD of results from triplicate measurements for each condition. (E) NanoLuc-tagged full-length HEV RNA produces infectious virus. Culture supernatants as well as cell lysates of Hep293TT cells electroporated with either B6Nluc or B6Nluc_GAD full-length RNA were collected at day 1 and day 3 as well as day 5 postelectroporation and used to infect naive HepG2/C3A cells, followed by determination of focus-forming units (ffu) to measure viral infectivity from intracellular or extracellular compartments as described in Materials and Methods. (F and G) Lysates from Hep293TT cells transfected with HEV83-2_B6Nluc or HEV83-2_B6Nluc_GAD RNA were used to infect naive HepG2/C3A cells. Infectivity was assessed by immunofluorescence 5 days postinfection using mouse MAb 1E6 against the ORF2 protein (F) or by luciferase assay (G). Cell nuclei were counterstained with DAPI (blue). The scale bar represents 20 μm. RLU levels were determined in triplicate 12-well plates measured twice each.

Efficient replication of HEV genomes harboring an HA tag in the ORF1 protein encouraged us to insert a small luciferase, i.e., NanoLuc (173 amino acids [approximately 19 kDa]), into the B6 site and to monitor HEV RNA replication by luciferase assay. The B6Nluc insertion was cloned into the full-length HEV83-2-27 construct, followed by *in vitro* transcription and electroporation of full-length viral RNA into Hep293TT cells (Fig. 8A). As shown in Fig. 8B, the presence of a full-length HEV RNA harboring NanoLuc at the B6 site allowed us to monitor replication over time. A replication-defective construct harboring a GAD mutation in the RdRp did not yield luciferase activity and ribavirin efficiently inhibited replication of the B6Nluc RNA construct. Analogous results were obtained with the B2Nluc construct (data not shown). Similar to the HA tag (data not shown), the B6Nluc insertion was stably maintained over at least 25 days including 5 cell passages (Fig. 8C).

The effective results from electroporation studies led us to examine whether the use of B6Nluc would allow the production of infectious virus. As shown in Fig. 8D, ELISA for HEV ORF2 protein in culture supernatants collected from full-length B6Nluc RNA-transfected Hep293TT cells revealed a time-dependent increase in capsid release. Moreover, infectivity as assessed by focus formation assay from intracellular and extracellular compartments showed a time-dependent increase, reaching titers of about 10^2^ focus-forming units (FFU) per ml, while no infectious virus was produced by cells transfected with the B6Nluc_GAD RNA as a negative control (Fig. 8E). In addition, HepG2/C3A cells were inoculated with lysates from Hep293TT cells transfected with B6Nluc or B6Nluc_GAD RNA and analyzed by immunofluorescence for the ORF2 protein and by luciferase assay. As shown in Fig. 8F and G, ORF2 protein was readily detected by immunofluorescence and significant luciferase activity was observed at day 5 postinfection. These results demonstrate that the insertion of functional NanoLuc was tolerated in the B6 site and was compatible with the production of infectious virus.

## DISCUSSION

Although HEV infection is becoming increasingly recognized, knowledge of the molecular virology and pathogenesis of hepatitis E remains incomplete. Important aspects of the viral life cycle are still poorly understood, in particular, the structure and function of ORF1 protein, i.e., the viral replicase, its processing or the lack thereof, and cellular factors involved in viral replication (7–9). The development of infectious clones enabling the study of HEV replication or detection of ORF1 protein in authentic systems remains difficult due to low expression levels and/or the limited sensitivity of currently available antibodies. Therefore, we made use of transposon-mediated random insertion coupled with selection of viable insertions in a replicon system to develop functional tagged HEV genomes allowing reproducible visualization of the ORF1 protein and monitoring of ORF1 expression in authentic infection and replication systems.

Analyses of ORF1 protein in heterologous expression systems revealed the protein to be membrane associated and to partially colocalize with markers of the ER and the ER-Golgi intermediate compartment, suggesting that HEV uses modified early secretory pathway membranes for replication (23). Although the antibodies used in these studies enabled the detection of ORF1 protein domains or of the full-length protein in overexpression systems, ORF1 protein detection in genuine infection and replication systems *in vitro* or in liver biopsy specimens from patients with hepatitis E has been difficult (12, 13).

Functional tagging of viral proteins has been widely used. However, positive-strand RNA viruses have evolved with respect to optimizing the size of their genomes and the multifunctionality of the encoded proteins; hence, they often do not tolerate tag insertions. In order to identify viable insertion sites in the HEV ORF1 protein, we exploited MuA transposase-mediated *in vitro* transposition to create random insertions within the ORF1 coding region of a selectable subgenomic HEV replicon derived from HEV genotype 3 clone 83-2-27, which does not harbor any naturally acquired insertion (15). In fact, the commonly used Kernow-C1/p6 clone harbors a 174-nucleotide insertion encoding 58 amino acids of human ribosomal protein S17 in the HVR (16).

Transposon-mediated random insertion has been pursued in studies of other positive-strand RNA viruses such as hepatitis C virus (24), Zika virus (25), dengue virus (26, 27), and Venezuelan equine encephalitis virus (28), providing tools for live-cell imaging and further insights into the respective viral life cycles (29, 30).

Our screen led to the identification of 8 viable insertion sites within ORF1, 1 located downstream of the methyltransferase domain, 6 located in the HVR, and 1 located between the helicase and RdRp domains. Remarkably, these sites mapped to the boundaries of functional ORF1 domains, with a strong concentration in the HVR, which represents the genetically most variable region of ORF1. As expected, no viable insertions were identified within the functional domains, i.e., the methyltransferase, helicase, or RdRp. Insertions in the HVR were better tolerated than insertions downstream of the methyltransferase and between the helicase and RdRp domains, allowing functional tagging with an HA epitope or a small luciferase. Immunofluorescence and immunoblot analyses or luciferase activity measurement allowed the efficient detection of ORF1 protein in cells replicating HA-tagged or NanoLuc-tagged subgenomic HEV replicons or full-length genomes. Moreover, full-length genomes harboring these tags in the HVR allowed the production of infectious virus, demonstrating the capacity of the identified sites to tolerate insertions without disabling virus production.

The results obtained in our random insertion screen point to the remarkable flexibility of the HVR. In this context, it is interesting that naturally acquired insertions in this region of the ORF1 protein have been previously observed in immunocompromised patients with chronic hepatitis E (18, 31–34). These insertions were either duplications of HEV sequences or host-derived sequences such as ribosomal sequences or other protein sequences. The lengths of natural insertions identified so far range from 117 to 333 nucleotides, i.e., from 39 to 111 amino acids, longer than the HA epitope sequences (57 nucleotides or 19 amino acids), whose insertion did not affect replication efficiency in our study, and shorter than the NanoLuc sequences (534 nucleotides or 178 amino acids), explaining the lower replication efficiency of the constructs harboring this reporter (data not shown). Of note, some of the natural insertions identified in patients with chronic hepatitis E provided a replication advantage in cell culture (16, 18, 31, 32); however, we did not observe any such advantage with the transposon or tag insertions examined in our study. Interestingly, the insertion site mapped between the helicase and RdRp domains corresponds to a natural insertion of 93 nucleotides or 31 amino acids found in rabbit HEV (genotype 3ra) (35, 36).

HEV replication sites have been poorly defined to date, and characterization of their replication complexes has been elusive. In our study, ORF1 protein was found to be associated with membranes and to colocalize with HEV RNA, pointing to candidate replication sites. These colocalized best with the tetraspanins CD63 and CD151 as markers of exosomes and multivesicular bodies and to only a very minor degree with ERGIC-53 and GM130 as markers of the ER-Golgi intermediate compartment and the Golgi apparatus, respectively. However, there was no notable colocalization with PDI and MAVS as markers of the ER and of mitochondria, respectively.

Interactions of ORF3 protein with tumor susceptibility gene 101 (TSG101) and the exosomal pathway have been reported previously to be required for HEV egress (37–39). In addition, Nagashima et al. reported CD63 and CD81 to be associated with quasi-enveloped HEV, suggesting hijacking of exosomal pathway components during viral egress (40). Hence, our findings suggest a close connection between HEV replication and assembly sites, as also supported by the partial colocalization of ORF1 with ORF2 and ORF3 proteins. Indeed, the involvement of replicase components in viral assembly has been well described for other positive-strand RNA viruses (41). In addition, or as an alternative, exosomal membranes may be required for establishment of the viral replication complex. Further studies, including studies employing electron microscopy, will be required to determine the structure and composition of the HEV replication complex.

The use of recombinant HEV harboring a NanoLuc insertion in the ORF1 protein allowed monitoring of viral replication over time in both transfection and infection settings and measurement of ORF1 expression in the context of a complete viral life cycle. In contrast to the use of a commonly used subgenomic replicon carrying a Gaussia luciferase reporter gene in place of ORF2 (16), luciferase activity in our system directly reflects replication and translation of the full-length viral RNA rather than the subgenomic RNA encoding the ORF2 and ORF3 proteins. Such constructs represent promising tools for the screening of novel antivirals, as shown in our study performed with ribavirin as a proof of concept.

In conclusion, transposon-mediated random insertion coupled with selection in a replicon system allowed the identification of viable insertion sites in the HEV ORF1 protein. HEV genomes harboring an epitope tag or NanoLuc in the HVR were found to be fully functional and to allow the production of infectious virus. NanoLuc allowed quantitative monitoring of HEV infection and replication by luciferase assay. HA-tagged replicons and full-length genomes allowed localization of putative sites of HEV RNA replication by the simultaneous detection of ORF1 protein by immunofluorescence and of viral RNA by FISH. Candidate HEV replication complexes were found in the cytoplasm at a site which partially overlaps ORF2 and ORF3 proteins as well as exosomal markers. Hence, tagged HEV genomes should allow further investigation of the subcellular localization, ultrastructure, and composition of viral replication complexes.

## MATERIALS AND METHODS

### Cell lines and reagents.

Hep293TT human hepatoblastoma cells (42) (kindly provided by Gail E. Tomlinson, University of Texas Health Science Center at San Antonio, San Antonio, TX) were cultured in RPMI 1640 medium supplemented with 10% fetal bovine serum (FBS) (both from Thermo Fisher Scientific, Waltham, MA). HepG2 human hepatoblastoma-derived subclone HepG2/C3A (17, 18) was obtained from the American Type Tissue Collection and was cultured in Dulbecco’s modified Eagle medium (Thermo Fisher Scientific) supplemented with 10% FBS.

### Plasmids.

All constructs described here were prepared using genotype 3 infectious clone HEV83-2-27 as a template (15) (pUC-HEV83-2 kindly provided by Koji Ishii and Takaji Wakita, National Institute of Infectious Diseases, Tokyo, Japan). Primers used in this study are listed in Table 2. All constructs were verified by sequencing.

**TABLE 2:** List of primers used in this study

Primer name	Nucleotide sequence (5′ → 3′)
HEVgt3-4487-fd	CCCGTGGTTCCGTCCCATTG
HEVgt3-5897-rv	GATGCCTCAGTAGCCATGAT
HEVgt3-GAD-fd	GCCTTTAAGGGTGCTGATTCGGTGGTCCT
HEVgt3-GAD-rv	AGGACCACCGAATCAGCACCCTTAAAGGC
HEVgt3-Neo-rv	ACAGACGACGTGATTCAGAAGAACTCGTCAAGAAGG
HEVgt3-Gluc-rv	ACAGACGACGTGATTTAGTCACCACCGGCCCCCTTG
Nluc_NotI-fd	ATTATTGCGGCCGCAGTCTTCACACTCGAAGA
Nluc_NotI-rv	ATTATTGCGGCCGCCCCGCCAGAATGCGTTCGCACA
pUC-EcoRI_A-fd	GGCAGACCACGTATGTGGTCG
pUC-EcoRI_A-rv	GGAACGTCCTACATCGACAG
pUC-Bam_B-fd	ATGCACAGTGCCGACGGT
pUC-Bam_B-rv	TGCTAGGTTGGCCGTACGG
pUC-BsiWI_C-fd	GTGGCCTGGTTCGAGGCTA
pUC-BsiWI_C-rv	CGGAACTCATAGCAATGTGC

Subgenomic HEV replicon constructs harboring either a neomycin phosphotransferase (Neo) or a Gaussia luciferase gene (Gluc) were prepared by PCR amplification with primers HEVgt3-4487-fd and HEVgt3-Neo-rv or HEVgt3-Gluc-rv, respectively, followed by cloning into the AflII/BmgBI sites of pUC-HEV83-2, yielding pUC-HEV83-2_Neo or pUC-HEV83-2_Gluc.

DNA fragments corresponding to regions A, B, or C of the ORF1 sequence (see Fig. 1A) flanked by restriction sites EcoRI/BamHI, BamHI/BsiWI, or BsiWI/KpnI, respectively, and harboring an HA epitope-encoding sequence flanked by transposon sequences at the specific insertion sites (see Table 1) were synthesized by GenScript (Piscataway, NJ). Fragments were cloned into pUC-HEV83-2_Neo, yielding pUC-HEV83-2_Neo_A-HA, pUC-HEV83-2_Neo_B1-HA, pUC-HEV83-2_Neo_B2-HA, pUC-HEV83-2_Neo_B4-HA, pUC-HEV83-2_Neo_B6-HA, and pUC-HEV83-2_Neo_C-HA. Full-length HEV83-2 constructs harboring an HA epitope tag at the B2 site or the B6 site were generated by cloning of synthesized DNA fragments into the BamHI/BsiWI sites of pUC-HEV83-2, yielding to pUC-HEV83-2_B2-HA and pUC-HEV83-2_B6-HA.

Excision of the HA tag from the pUC-HEV83-2_Neo constructs by NotI digestion and religation yielded constructs harboring the original transposon sequence, i.e., pUC-HEV83-2_Neo_A, pUC-HEV83-2_Neo_B1, pUC-HEV83-2_Neo_B2, pUC-HEV83-2_Neo_B4, pUC-HEV83-2_Neo_B6, and pUC-HEV83-2_Neo_C.

The NanoLuc sequence was amplified from pTM-Nluc (43) (kindly provided by Sylvia Rothenberger, Institute of Microbiology, Centre Hospitalier Universitaire Vaudois, Switzerland) by using primers Nluc_NotI-fd and Nluc_NotI-rv primers, followed by cloning into the NotI site of pUC-HEV83-2_B6, yielding pUC-HEV83-2_B6Nluc. A GAD polymerase-defective construct, where an aspartate of the catalytic triad is mutated to alanine (GDD → GAD), was prepared by two-step overlap extension PCR First, PCR amplifications were performed using pUC-HEV83-2 as the template and HEVgt3-4487-fd or HEVgt3-GAD-fd as forward primer and HEVgt3-GAD-rv or HEVgt3-5987-rv as reverse primer. Second, overlap extension PCR was performed using the two amplicons as the template and primers HEVgt3-4487-fd and HEVgt3-5987-rv, followed by cloning into the AflII/BmgBI sites of pUC-HEV83-2_B6Nluc, yielding pUC-HEV83-2_B6Nluc_GAD.

### Antibodies.

Rabbit monoclonal antibody (MAb) C29F4 against the HA epitope was purchased from Cell Signaling Technology (Leiden, The Netherlands). Mouse MAbs against HEV ORF2 (1E6), MAVS (Adri-1), GM130 (clone 35), and β-actin (AC-15) were from Millipore (Burlington, MA), AdipoGen (San Diego, CA), BD Biosciences (San Jose, CA), and Sigma-Aldrich (St. Louis, MO), respectively. Mouse MAbs against ERGIC-53 (OTI108), PDI (1D3), and CLIMP63 (G1/296) were from Enzo Life Sciences (Farmingdale, NY), and MAbs against CD63 (MX491295) and CD151 (11G5a) were from Santa Cruz Biotechnology (Dallas, TX). Recombinant mouse antibody MRB198 against genotype 3 HEV ORF3 protein have been described previously (11). Alexa Fluor 488 and Alexa Fluor 694 anti-mouse IgG as well as Alexa Fluor 594 anti-rabbit IgG secondary antibodies were from Thermo Fisher Scientific. Horseradish peroxidase-conjugated anti-mouse IgG and anti-rabbit IgG secondary antibodies were from GE Healthcare (Chicago, IL) and Agilent Technologies (Santa Clara, CA), respectively.

### Transposon-mediated insertion screen.

Plasmid pUC-HEV83-2_Neo, coding for a selectable subgenomic HEV replicon, served as the template for random 15-bp sequence insertion using transposon-based technology following the manufacturer’s recommendation (mutation generation system kit; Thermo Fisher Scientific). In brief, pUC-HEV83-2_Neo was subjected to MuA transposase-mediated random insertion, followed by selection with kanamycin (50 μg/ml) and ampicillin (100 μg/ml). ORF1 DNA fragments harboring transposon insertions and designated fragment A (EcoRI/BamHI), B (BamHI/BsiWI), C (BsiWI/KpnI), or D (KpnI/PflFI) were cloned back into the parental pUC-HEV83-2_Neo construct. Sublibraries were digested with NotI and religated to excise the kanamycin resistance cassette inserted by the transposition reaction. As a consequence, 4 sublibraries of pUC-HEV83-2_Neo constructs harboring random 15-bp sequences in ORF1 were prepared and subjected to *in vitro* transcription, followed by electroporation into Hep293TT cells and selection with 500 μg/ml G418 (Life Technologies) for 12 days. G418-resistant cell populations were lysed and RNAs isolated by the use of a NucleoSpin RNA kit (Macherey-Nagel, Düren, Germany) following the manufacturer’s recommendations. Total RNA was reverse transcribed by random priming using a PrimeScript RT-PCR kit (TaKaRa Bio, Shiga, Japan), followed by PCR using primers pUC-EcoRI_A-fd and puC-EcoRI_A-rv (fragment A), pUC-Bam_B-fd and pUC-Bam_B-rv (fragment B), or pUC-BsiWI_C-fd and pUC-BsiWI_C-rv (fragment C). Amplified fragments were cloned into the pCR-BluntII-TOPO vector using a Zero Blunt PCR cloning kit (Thermo Fisher Scientific), followed by sequencing. Identified insertion sites are listed in Table 1.

### *In vitro* transcription and cell electroporation.

Capped HEV RNA was prepared after linearization of replicon and full-length constructs by the use of HindIII, followed by *in vitro* transcription and 5′ capping of the RNA using a mMESSAGE mMACHINE kit from Ambion (Thermo Fisher Scientific) according to the manufacturer's instructions. RNA was purified using a QIAamp viral RNA kit (Qiagen, Hilden, Germany) according to the manufacturer's instructions. A 5-μg volume of purified RNA was electroporated into 6 × 10^6^ Hep293TT cells by the use of a BTX ECM830 electroporator (250 V, 1 pulse, 3 ms, 2-mm gap cuvette) and Cytomix buffer, as described previously (44). Electroporated cells were seeded into either two 10-cm-diameter dishes for HEV production or two 12-well plates with glass coverslips for immunofluorescence analyses.

### Colony formation assay and crystal violet staining.

At 3 days postelectroporation of selectable subgenomic replicon RNA, cells were cultured with 500 μg/ml G418. Culture medium was changed every 2 to 3 days, and after 12 days antibiotic-resistant colonies were fixed in 7% formaldehyde, followed by staining with 1.25% crystal violet–25% ethanol to facilitate colony counting.

### Virus infection and determination of infectivity.

At 5 days postelectroporation of *in vitro*-transcribed full-length HEV RNA, culture supernatants and Hep293TT cells were harvested to determine infectivity in the extracellular and intracellular compartments, respectively. Cell pellets were subjected to three freeze-and-thaw cycles, followed by centrifugation for 15 min at 3,000 × *g* to prepare the intracellular sample. To inoculate HepG2/C3A cells seeded in a 24-well plate, a 1/10 volume of the total extracellular (200 μl) or intracellular (50 μl) virus sample was used. Infectivity was determined on HepG2/C3A cells by focus formation assay using anti-ORF2 antibody 1E6.

### Quantitative RT-PCR.

Total RNA was extracted from HEV-infected HepG2/C3A cells using a NucleoSpin RNA isolation kit (Macherey-Nagel, Oensingen, Germany) and served as the template for cDNA synthesis using a High Capacity cDNA reverse transcription kit (Applied Biosystems) following the manufacturer's instructions. Quantitative PCR was performed with HEV-specific primers and probes as described previously (45) using TaqMan Fast Universal PCR Master Mix and a StepOne Plus real-time PCR system (Applied Biosystems). Results were normalized to GAPDH (glyceraldehyde-3-phosphate dehydrogenase) RNA determined using the primers and probe from Applied Biosystems and expressed in relation to the parental genome. Each data point represents results from 3 different wells, each measured in duplicate.

### Immunoblotting.

Cells were lysed 5 days postelectroporation using Laemmli buffer. Protein lysates were subjected to sodium dodecyl sulfate-polyacrylamide gel electrophoresis (SDS-PAGE), followed by immunoblot analysis, as described previously (46).

### Immunofluorescence.

Electroporated cells were cultured on glass coverslips for 5 days and then fixed with 4% paraformaldehyde for 10 min at 20°C. Permeabilization was carried out by incubation in phosphate-buffered saline (PBS)–0.2% Triton X-100 for 10 min at 20°C. Subsequently, cells were blocked with 10% goat serum in PBS–0.05% Tween 20 for 30 min at 20°C, followed by successive incubations with primary antibodies for 12 h at 4°C and with Alexa Fluor secondary antibodies for 1 h at 20°C. Cell nuclei were counterstained with DAPI (4′,6-diamidino-2-phenylindole; Thermo Fisher Scientific). Slides were mounted in ProLong (Thermo Fisher Scientific) and examined under a Zeiss LSM 780 confocal laser scanning microscope. Images were processed and mean Pearson's correlation coefficients determined using ImageJ software based on ≥10 different cells.

### ELISA for HEV ORF2 protein.

ORF2 protein release was analyzed in 50 μl of supernatant collected from Hep293TT cells electroporated with parental or tagged full-length HEV genomes using Wantai HEV-Ag ELISA *Plus* (Wantai Biopharmaceuticals, Beijing, China) according to the manufacturer’s instructions. After stopping the final reaction, optical density (OD) at 450 nm was measured with a Multiscan Ascent ELISA plate reader (Thermo Fisher Scientific). Each condition was measured in triplicate, and the resulting OD values were corrected by subtraction of the OD value determined for a blank medium sample.

### Fluorescence *in situ* hybridization (FISH).

All reagents were purchased from Advanced Cell Diagnostics (Newark, CA), and FISH was performed using an RNAscope fluorescent multiple kit according to the manufacturer’s instructions. In brief, Hep293TT cells grown onto glass coverslips were fixed 5 days postelectroporation with 10% formaldehyde for 30 min at 20°C, washed with PBS, and incubated for 10 min at 20°C in Protease III buffer. Cells were subsequently washed four times with PBS and subjected to hybridization with probe V-HEV-C2 (ACD catalog no. 468111) diluted 1:50 for 2 h at 40°C. Specimens were then washed with PBS and incubated successively with amplification solutions Amp 1-FL (30 min at 40°C), Amp 2-FL (15 min at 40°C), Amp 3-FL (30 min at 40°C), and Amp 4-FL (15 min at 40°C). The V-HEV-C2 probe, composed of 120 ZZ pairs targeting nucleotides 19 to 7257 of the HEV genome, was designed by the manufacturer on the basis of a consensus HEV sequence. Following FISH, cells were blocked with 10% goat serum and processed for immunofluorescence as described above.

### Membrane flotation assay.

At 5 days postelectroporation with full-length HEV RNA, Hep293TT cells were subjected to Dounce homogenization in hypotonic buffer (10 mM Tris-HCl [pH 7.4], 2 mM MgCl_2_) containing a protease inhibitor cocktail (Roche Diagnostics, Penzberg, Germany). After elimination of nuclei by centrifugation at 1,200 × *g* for 10 min, the supernatants were left untreated or were treated with 1% Triton X-100 for 20 min on ice. Samples were then subjected to equilibrium centrifugation (100,000 × *g*, 4°C, 20 h) in 5% to 37.5% (wt/vol) Nycodenz (Axis Shield, Oslo, Norway) gradients using an Optima Max-XP Ultracentrifuge (Beckman Coulter; Brea, CA). Subsequently, the fractions were collected from the top, and equal volumes were subjected to immunoblotting.

### Luciferase assays.

Nanoluciferase activity was measured in cells electroporated with full-length HEV83-2_B6-Nluc or HEV83-2_B6Nluc_GAD RNA using a Nano-Glo luciferase assay system (Promega, Madison, WI) according to manufacturer's recommendation. Cells were lysed in 100 μl Nano-Glo buffer, followed by the addition of 50 μl Nano-Glo substrate solution to 50 μl of lysate. Luciferase activities were measured twice in 3 different wells at each time point using a Berthold luminometer (Bad Wildbad, Germany). Inhibition of replication was assessed by treatment with 50 μM ribavirin (Sigma-Aldrich).

### Statistical analyses.

Significance values were calculated by using the unpaired *t* test with the GraphPad Prism 7 software package (GraphPad Software, San Diego, CA).

### Data availability.

Raw data have been deposited in the Zenodo repository (https://doi.org/10.5281/zenodo.3341480).
